# Surgical systematic reviews: best available evidence or disposable waste?

**DOI:** 10.1515/iss-2022-0029

**Published:** 2024-07-16

**Authors:** Rosa Klotz, Solveig Tenckhoff, Pascal Probst

**Affiliations:** Department of General, Visceral and Transplantation Surgery, Heidelberg University Hospital, Heidelberg, Germany; Study Center of the German Society of Surgery, Heidelberg University Hospital, Heidelberg, Germany; Department of Surgery, Cantonal Hospital Thurgau, Frauenfeld, Switzerland

**Keywords:** systematic reviews, evidence-based medicine, RCT, SR

## Abstract

Evidence-based medicine demands treatment options for patients to be based on the current best available evidence. Systematic reviews (SRs) with meta-analyses allow surgeons to make therapeutical decisions in accordance with the highest level of evidence. Also, high-quality SRs support physicians to challenge the colossal amount of new research data created daily. The systematic review working group of the Study Center of the German Society of Surgery (SDGC) has created specific methodological literature regarding surgical SRs, giving recommendations to assess critical risk of bias and to prevent the creation of SRs that do not provide any new insights to the field. SRs should only be considered if there is new clinically relevant data available that allows the SR to create novel evidence. To address the dilemma of new SRs generated without adding new evidence, living systematic reviews and evidence mapping represent an innovative approach, in which SRs are regularly updated with new research data.

## Introduction

The identification of the optimal treatment option based on the best available scientific data while integrating personal medical experience and the individual patient’s needs is known as evidence-based medicine (EBM) [1]. Randomized controlled trials (RCTs) and systematic reviews (SRs) with meta-analyses that consider these RCTs enable the highest level of evidence. Besides, an evidence-based approach is compulsory to minimize irrelevant, nonscientific clinical research [2].

In the past few decades, medicine has experienced an information explosion with almost uncontrollable amounts of data from scientific research. Surgeons are faced with the challenge of integrating this rapidly growing knowledge into clinical practice and thus into patient care. Since time is a limited resource, it is hardly possible to keep track of the new data every day. Twenty-five years ago, only 25 % of surgical interventions were based on RCTs [3]. Current data regarding this association are lacking [4]; however, large parts of current guidelines are still based on low level evidence.

High-quality SRs are, therefore, of great importance in the healthcare system. Besides, they are the basis for the development of clinical guidelines, can identify research gaps, and provide recommendations for future clinical trials.

**Figure 1:**
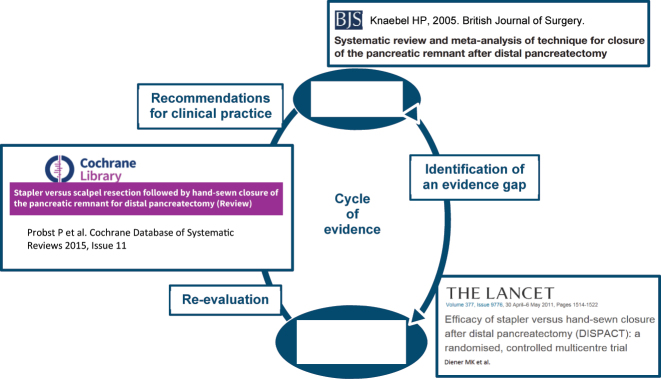
Cycle of evidence illustrated by the example of comparison of two closure techniques of the pancreatic remnant after distal pancreatectomy.

The systematic review working group of the Study Center of the German Society of Surgery (SDGC) was founded in 2005. This working group has created specific methodological literature regarding surgical SRs [5], [6], [7], [8], [9], disseminates the expertise required to conduct SRs among German surgeons, and supports them throughout this process. More than 80 SRs were published. Selected SRs completed the cycle of evidence, some of them in combination with RCTs performed by the SDGC. Figure 1 illustrates this cycle with an example based on the DISPACT trial [10], 11] and related SRs done by the SDGC.

## Best available evidence

SRs are considered original research in most journals, especially when a meta-analysis is performed [12], 13]. To prevent the classical “garbage in, garbage out” phenomenon systematic reviews in the field of surgery must address some specific challenges. Among others, the following published recommendations should be considered [6]:

1. When formulating a research question, a well-focused and answerable question according to the PICOS criteria (Patient, Intervention, Comparison, Outcome, and Study design) is mandatory. In a SR, nonrandomized studies should be included only for specific reasons, such as rareness of the indication and nonavailability of data from randomized trials.
2. When describing the intervention and the comparison (control), it is important to define and standardize the exact procedure or group of relevant procedures to guarantee comparability and to ensure that if interventions are deemed effective, they can actually be reproduced and implemented in clinical practice. This is particularly relevant in evaluation of complex surgical procedures. The control group must be carefully selected in order to minimize bias. That can occur, for example, if the treatment in the control group is outdated already [14].
3. Assessing a primary study’s methodological quality is obligatory to ensure that the quantitative merit of a study can be interpreted. Specifics in surgical trials include that the type of blinding, industry bias, and experience of the surgeon regarding the intervention should be addressed and described.
  – Blinding in surgical trials is challenging. “Double-blinding” is not reasonably transferable to all surgical trials, but it is recommended to report if study contributors (patient, surgeon, outcome assessor, or data analyst) were blinded and whether endpoints might be biased by nonblinding [9].
  – Funding by industry is a definitely interesting topic: in general and abdominal surgical trials industry, funding leads to exaggerated positive reporting of outcomes [8]. A systematic review and meta-analysis evaluating the outcomes of robotic surgery showed that financial sponsorship by industry appears to be associated with a higher likelihood of studies reporting a benefit of robotic surgery. These findings suggest a dollar amount where financial payments influence reported clinical results [15]. Thus, conflict of interest reporting is mandatory both in primary studies and SR.
  – Also, surgeon’s experience and learning curves are of high relevance when evaluating the risk of bias of a primary study, which should be included in a SR. In minimally invasive surgery, it takes longer than in open surgery to complete a learning curve. Besides, the relevant parameters (e.g., duration of surgery, blood loss, conversion rates, postoperative complications) based on which one considers the learning curve as completed are heterogenous [16].
  – Surgical studies in particular are subject to reporting bias, as they are often terminated prematurely for futility or the primary endpoint is changed during the course of the study [17]. In a systematic review including RCTs published in 2009 and 2010 in 10 high-impact factor surgical journals, 30 % of all trials showed discrepancies between the initially registered and the finally published primary outcome. Furthermore, the discrepancy favored a statistically significant primary outcome in >91.7 %.

Accordingly, options for blinding, funding by industry and surgeon’s expertise, and learning curves should be included in critical risk of bias assessment.

## Disposable waste: mechanisms and examples

**Figure 2:**
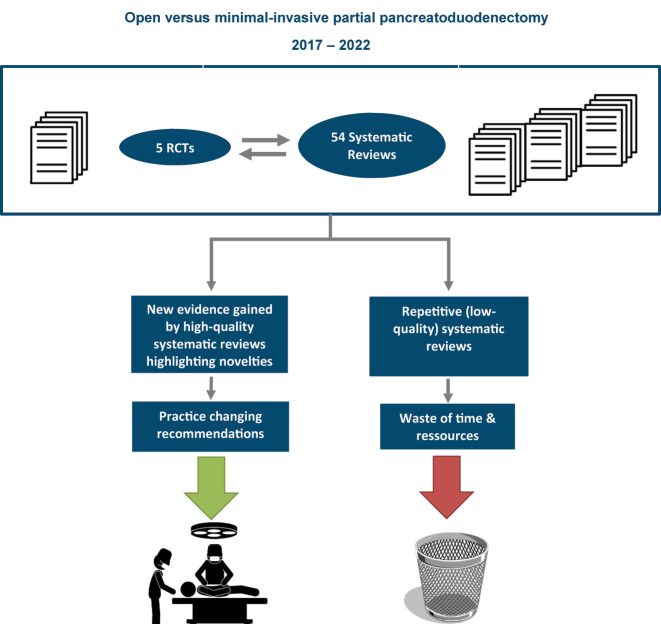
Creating evidence vs. waste by the example of open vs. minimal-invasive partial pancreatoduodenectomy.

In surgical care, there are megatrends that are accompanied by scientific publications leading to redundant publications. For many journals, this mechanism is a huge driver of economic success. For the publishing researchers to publish also means success. Superficially, this is a win-win situation. However, clinicians looking for best available evidence are the losers of this mechanism. For example, minimal-invasive partial pancreatoduodenectomy has become a megatrend among pancreatic surgeons and over 5 years (2017–2022), five RCTs were published. In the same time, 54 SRs were published and most of them did not create novel insights although their creation consumed resources and time (Figure 2). Another example for redundant systematic reviews is the topic single incision for laparoscopic colectomy: Overall, two RCTs compared single-incision vs. multi-incision laparoscopic approaches, but the topic was dealt within six meta-analyses [18].

Unfortunately, SRs are sometimes seen as simple, quick publications, but should only be performed when there are clinically relevant new studies so that novel evidence can actually be generated. In fact, performing research at high evidence levels, e.g., by conducting a SR to identify open research questions followed by a multicenter RCT and conduction of another SR to pool and interpret the new data with the existing ones is a long-lasting, expensive business.

## Innovative approaches: the evidence map and living systematic reviews

Novel approaches have been developed in the past few years to avoid redundant publications like living systematic reviews and evidence mapping. However, the traditional way of publishing within a journal article hampers their development. Unlocking its true potential is only possible by combining online availability and rigorous scientific methods. Then, evidence-based answers based on primary scientific data on a particular medical question can be found quickly and easily. Here as an example, the “Evidence Map of Pancreatic Surgery” provided by the ISGPS [19] is a new, helpful tool for clinicians to find relevant literature completely and clearly in pancreatic surgery. The map provides “EVIdence at a glance,” which is updated in a continuous way compiled by the same methods used for a high-quality standalone systematic review. The evidence map is available via www.evidencemap.surgery and their creators now plan to provide more maps via EVIglance.com.

In summary, a living SR is a SR that is regularly revised with new research data. By compiling findings of selected studies on a specific research topic, they include all new information and so is an efficient and trustworthy tool to continually be up to date and point out gaps in primary research. In the last few years, the variety of living SR that considered the latest evidence regarding the COVID pandemic and SARS-COV2 virus gives examples for usefulness of those tools.

## Conclusions

Using the best available evidence is prerequisite for optimal treatment decisions with patients. But adding new evidence to a field of research via performance of a SR is only warranted if there are open research questions, which on the one hand are investigated by minimum two primary studies provided that their results can be pooled in a meaningful way but on the other hand are not yet answered by an already published SR. Only under these conditions, a methodologically sound SR with evaluation of existing clinical data in accordance with previously published recommendations can close evidence gaps.

Conversely, answering questions by another SR without availability of new primary data will produce disposable waste. Reasons for this phenomenon include that publications are of utmost need for academic staff to step up the “career ladder” and SRs are considered low hanging fruits.
